# Voltage Gated Calcium Channels Negatively Regulate Protective Immunity to *Mycobacterium tuberculosis*

**DOI:** 10.1371/journal.pone.0005305

**Published:** 2009-04-23

**Authors:** Shashank Gupta, Nasir Salam, Varsha Srivastava, Rupak Singla, Digamber Behera, Khalid U. Khayyam, Reshma Korde, Pawan Malhotra, Rajiv Saxena, Krishnamurthy Natarajan

**Affiliations:** 1 Immunology Group, International Centre for Genetic Engineering and Biotechnology, New Delhi, India; 2 Department of TB & Respiratory Diseases, LRS Institute of TB & Respiratory diseases, New Delhi, India; 3 Malaria Group, International Centre for Genetic Engineering and Biotechnology, New Delhi, India; 4 School of Life Sciences, Jawaharlal Nehru University, New Delhi, India; University of Hyderabad, India

## Abstract

*Mycobacterium tuberculosis* modulates levels and activity of key intracellular second messengers to evade protective immune responses. Calcium release from voltage gated calcium channels (VGCC) regulates immune responses to pathogens. In this study, we investigated the roles of VGCC in regulating protective immunity to mycobacteria in vitro and in vivo. Inhibiting L-type or R-type VGCC in dendritic cells (DCs) either using antibodies or by siRNA increased calcium influx in an inositol 1,4,5-phosphate and calcium release calcium activated channel dependent mechanism that resulted in increased expression of genes favoring pro-inflammatory responses. Further, VGCC-blocked DCs activated T cells that in turn mediated killing of *M. tuberculosis* inside macrophages. Likewise, inhibiting VGCC in infected macrophages and PBMCs induced calcium influx, upregulated the expression of pro-inflammatory genes and resulted in enhanced killing of intracellular *M. tuberculosis*. Importantly, compared to healthy controls, PBMCs of tuberculosis patients expressed higher levels of both VGCC, which were significantly reduced following chemotherapy. Finally, blocking VGCC in vivo in *M. tuberculosis* infected mice using specific antibodies increased intracellular calcium and significantly reduced bacterial loads. These results indicate that L-type and R-type VGCC play a negative role in *M. tuberculosis* infection by regulating calcium mobilization in cells that determine protective immunity.

## Introduction

*Mycobacterium tuberculosis^5^* (*M. tuberculosis*) mediated mortality and morbidity continue to rise with the emergence of extremely-drug resistant bacteria, co-infection with HIV and variable efficacy of protection offered by *M. bovis* Bacillus Calmette-Guerin (BCG) vaccine [1], [2]. Therefore, there is a need to elucidate factors that regulate protective immune responses against this pathogen and to identify targets for development of new drugs [3]. Macrophages, dendritic cells (DCs) and T cells are instrumental in mediating immunity to mycobacteria [4]–[7]. Though, macrophages serve as long-term hosts for *M. tuberculosis* [4], [5], *M. tuberculosis*, DCs are also infected by *M. tuberculosis* and are crucial to initiate protective immune responses [8].

Calcium is a universal and important ion that plays an obligatory role in the regulation of a number of cellular processes. Calcium concentrations and oscillations govern the selective activation and inactivation of transcription factors [9]. In most cells, a typical calcium response occurs in two phases [10]. The initial response is the depletion of intracellular stores from the endoplasmic reticulum (ER). This is followed by the activation of store operated calcium channels that leads to a sustained increase in intracellular calcium concentrations [11]. This second phase of calcium influx is either via calcium release calcium activated (CRAC) channels or via Voltage Gated Calcium Channels (VGCC) or both [12]. The VGCC consist of a transmembrane alpha subunit along with a cytoplasmic beta subunit that mediates signal transduction, with the gamma and delta subunits completing the core complex [13]. Several intracellular proteins and adaptors show close associations with VGCC subunits and regulate various cellular processes [14].

Calcium plays a determinant role in the generation of pro-inflammatory responses [15] and also regulates the survival of mycobacteria in macrophages. Calcium dependent phagosome maturation involves mycobacterial inhibition of sphingosine kinase that directly contributes to survival of *M. tuberculosis* within human macrophages [16]. In addition, tuberculosis toxin has been shown to inhibit phagosome maturation that involves the calmodulin-PI3K hVPS34 cascade [17]. Further, L-type VGCC has been shown to play major roles in regulating calcium homeostasis in lysosomal storage disease [18] and in *Legionella pneumophila* infection [19].

We had earlier shown that several *M. tuberculosis* antigens including culture filtrate protein (CFP)-10 (also known as MTSA-10) induce the differentiation and maturation of DCs [reviewed in 20],[21]. CFP-10 differentiated DCs (CFP10-DCs) are phenotypically and morphologically similar to DCs differentiated conventionally with GM-CSF [20]. However, functional characterization showed that, unlike GM-CSF-DCs that induce pro-inflammatory responses, CFP10-DCs induce suppressor responses [22]. Further, CFP10-DCs mount poor oxidative burst that results in increased bacterial burden [23]. Supplementing calcium results in increased oxidative burst and reduces bacterial loads. In addition, we recently showed that mycobacteria infected CFP10-DCs show reduced secretion of pro-inflammatory chemokines and cytokines. Conditioning CFP10-DCs with either RANTES & IP-10 or with IL-12 & IFN-γ results in increased mobilization of intracellular calcium and the induction of pro-inflammatory responses [24]. This in turn leads to increased clearance of established *M. tuberculosis* infection in mice which was better than that observed with drug treatment.

Since calcium played an important role in our experiments and as the role of VGCC in mediating calcium mobilization during *M. tuberculosis* infection has not been investigated in detail, we therefore, investigated the roles of L-type and R-type VGCC during *M. tuberculosis* infection. Since CFP10-DCs and GM-CSF-DCs share phenotypic similarities [21]) but differed in their functional outcomes [22], [23], we carried out parallel experiments with both DCs. This approach not only brings out mechanistic differences between the two DCs but also highlights the functional relevance of DC differentiation by *M. tuberculosis* antigens such as CFP-10. Our data show that inhibiting L-type and R-type VGCC in DCs, macrophages and PBMCs increases calcium influx. This results in enhanced expression of pro-inflammatory genes that play critical roles in protective immunity. Importantly, blocking L-type and R-type VGCC in macrophages and PBMCs results in reduced burden of virulent *M. tuberculosis*, blocking L-type and R-type VGCC in DCs, activates T cells that mediate killing of *M. tuberculosis* inside macrophages. PBMCs of patients with active TB disease express high levels of the two VGCC. Significantly, injecting antibodies to L-type and R-type VGCC in mice carrying an established *M. tuberculosis* infection results in reduced bacterial burden. Together, our results suggest a positive correlation of the expression levels of these VGCC with severity of TB disease and indicate that L-type and R-type VGCC could be potential therapeutic targets for treating tuberculosis.

## Results

### Inhibiting L-type and R-type VGCC in DCs increases calcium influx

**Figure 1:**
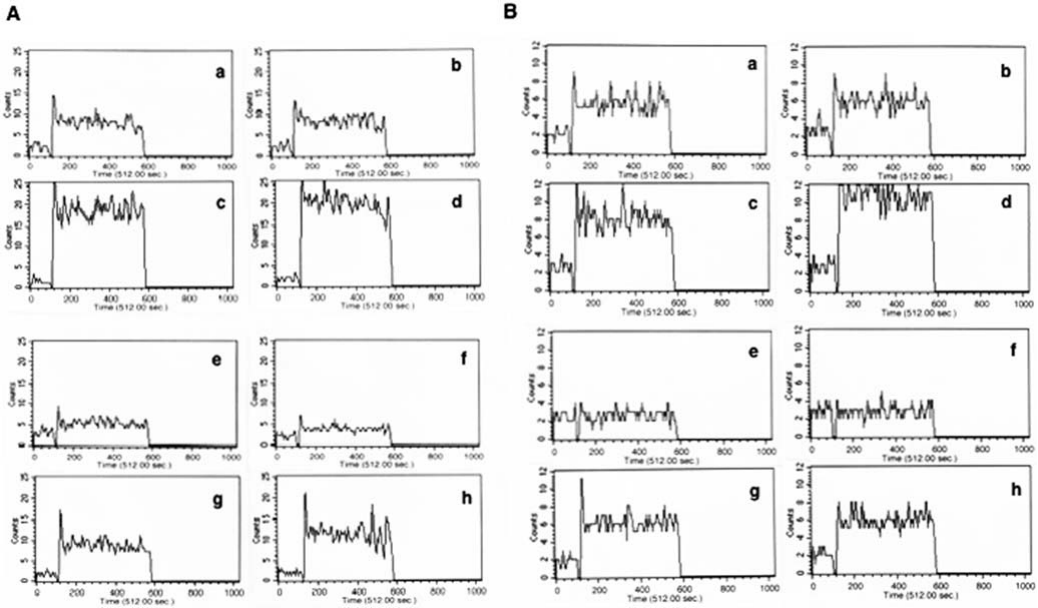
Neutralizing L-type and R-type VGCC in DCs induces calcium influx. Real time increase in calcium influx over 5 min in DCs stimulated with 1 MOI BCG. A, GM-CSF-DCs (a–d) or CFP10-DCs (e–h). Prior to stimulation, DCs were blocked with- anti-L-type antibody (c & g) or anti-R-type antibody (d & h) or non-specific antibody (b & f). B, calcium influx in BCG stimulated GM-CSF-DCs (a–d) and CFP10-DCs (e–h) following siRNA mediated silencing of L-type (c & g), R-type (d & h). siRNA against firefly luciferase was used as non-specific control (b & f). Data are representative of three to five independent experiments.

As mycobacteria induced calcium influx was superior in GM-CSF-DCs than in CFP10-DCs [23], [24], to begin with looked at the roles of L-type and R-type VGCC to investigate their role in calcium mobilization and the effects thereof on immunity to and survival of mycobacteria. Although biopharmacological inhibitors to L-type and R-type VGCC are available [25], in our hands these inhibitors were toxic to cells even at 0.5×IC_50_ concentrations and hence could not be used. Therefore, we used specific antibodies to L-type and R-type VGCC in our experiments. At the onset, we ensured that the above antibodies showed binding to DCs by FACS (Figure S1). Our results clearly show that antibodies to both L-type (panels a and d) and R-type VGCC (panels b and e) showed binding to VGCC on CFP10-DCs (panels a–c) and GM-CSF-DCs (panels d–f), while incubation with non-specific antibody (panels c and f) showed insignificant binding. We next analyzed the effect of incubation of DCs with these antibodies on calcium influx upon BCG stimulation. As shown in Figure 1A, stimulation of GM-CSF-DCs (panel a) resulted in a robust (164 µM) influx of calcium, while stimulation of CFP10-DCs (panel e) induced weak (78 µM) calcium mobilization. Surprisingly, incubation with either L-type (panel c & g) or R-type (panel d & h) VGCC resulted in a significant increase in calcium influx in both GM-CSF-DCs (536 and 598 µM). A similar increase in calcium influx was observed in CFP10-DCs (240 and 259 µM). Incubation with a non-specific antibody (panel b & f) had no significant effect. In addition, incubation with anti-L-type or anti-R-type antibody had no effect on calcium levels in uninfected cells (data not shown). Similar results were obtained when DCs were stimulated with *M. tuberculosis* H37Rv whole cell lysate instead of BCG indicating that the increase in calcium obtained upon blocking L-type and R-type VGCC was not related to the virulence of the strain (data not shown). It has been shown CFP-10 forms a dimer with another *M. tuberculosis* specific antigen, Early Secreted Antigenic target of 6 kDa (ESAT6) [26]. We therefore, differentiated DCs with CFP10:ESAT6 dimer and observed that similar to CFP10-DCs, blocking L-type and R-type VGCC in DCs differentiated with CFP-10:ESAT6 dimer also increased calcium influx (data not shown).

In order to see if incubation with antibodies resulted in either blocking or stimulation of VGCC, we did a similar experiment employing siRNAs against L-type and R-type VGCC. Figure S2 shows that siRNA treatment indeed decreased the mRNA levels of L-type and R-type VGCC in CFP10-DCs and GM-CSF-DCs while treatment with non-specific siRNA had no significant effect with respect to the respective controls. Next, as shown in Figure 1B, inhibiting L-type (panels c and g) or R-type (panels d and h) VGCC with specific siRNA resulted in a similar increase in calcium mobilization, while non-specific siRNA had no effect (panels b and f). This indicated that the observed effects with antibody incubation were a result of blocking of the VGCC. This further indicated that L-type and R-type VGCC play an inhibitory role in mobilizing calcium influx in DCs.

We also looked at calcium influx using live cell imaging by time-lapse confocal video-microscopy. However, over here, experiments were conducted with *M. tuberculosis* whole cell lysate (WCL) instead of live mycobacteria. Our previous results have demonstrated that CFP10-DCs induce similar responses to *M. tuberculosis* WCL and live mycobacteria [23]. Stimulation of CFP10-DCs with *M. tuberculosis* WCL induced a weak increase in calcium influx in the cells (Movie S1). Blocking L-type (Movie S2) or R-type (Movie S3) VGCC increased calcium influx as seen from increased fluorescence of a number of cells and appearance of new fluorescing cells. Similar results were obtained with GM-CSF-DCs (data not shown). Figure S3 graphically represents the data obtained by video microscopy. Blocking L-type (red line) or R-type (green line) VGCC in CFP10-DCs induced a significant increase in calcium influx upon *M. tuberculosis* WCL stimulation.

### CFP10-DCs express higher levels of L-type and R-type VGCC

**Figure 2:**
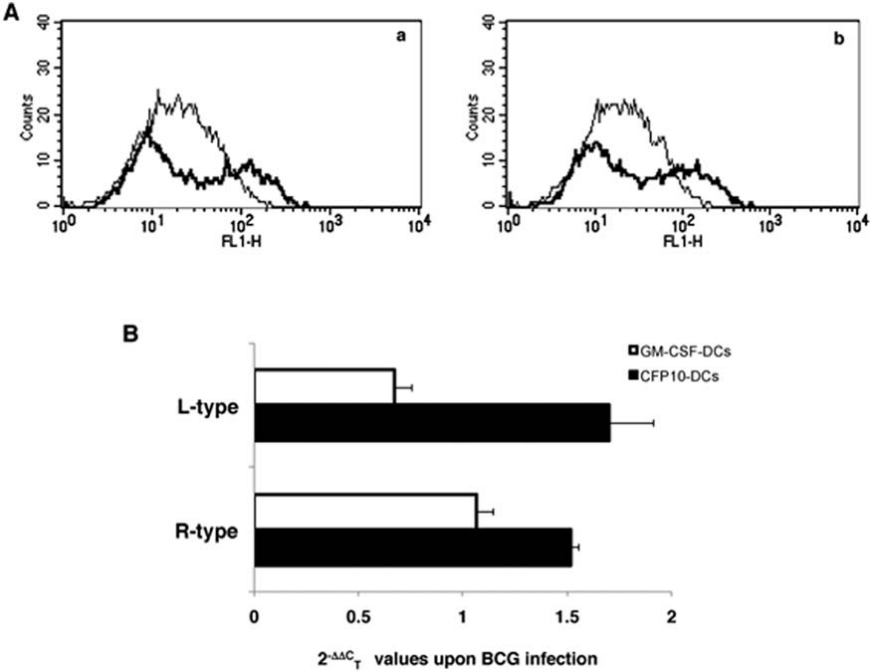
CFP10-DCs express higher levels of L-type and R-type VGCC following BCG infection. Histograms in Panel A show surface levels of L-type (panel a) and R-type (panel b) in BCG infected CFP10-DCs (thick lines) and BCG infected GM-CSF-DCs (thin lines). Panel B shows, results from qPCR of L-type and R-type VGCC transcripts in BCG infected DCs. The Bars represent 2^−ΔΔC^_T_ values in BCG infected CFP10-DCs and GM-CSF-DCs. Data are the mean of three independent experiments. Error bars represent mean±S.D. P<0.008 (for L-type levels between CFP10-DCs and GM-CSF-DCs); P<0.02 (for R-type levels between CFP10-DCs and GM-CSF-DCs). Two-tailed Student's t-test was employed for P values.

Since blocking L-type and R-type VGCC increased calcium influx in both GM-CSF-DCs and CFP10-DCs, we investigated whether the differential calcium mobilization observed in CFP10-DCs and GM-CSF-DCs was a result of differential expression of L-type and R-type VGCC in the two DCs. The binding data in Figure S1 indicated that uninfected CFP10-DCs expressed higher surface levels of both L-type and R-type VGCC when compared with uninfected GM-CSF-DCs. This was also reflected in the transcript levels of the two DCs (Figure S2). We extended these observations by looking at their levels following infection of DCs with BCG. As shown in Figure 2A, both L-type (panel a) and R-type (panel b) VGCC levels in BCG infected CFP10-DCs were significantly higher when compared with BCG infected GM-CSF-DCs. We further confirmed this by looking at their transcript levels by qPCR. As shown in Figure 2B, BCG infected CFP10-DCs expressed significantly higher levels of L-type and R-type VGCC mRNA (P<0.04 and P<0.05, respectively), when compared with BCG infected GM-CSF-DCs. These results indicated that poor calcium influx in CFP10-DCs in response to mycobacterial stimulation could be a result of increased expression of L-type and R-type VGCC.

### Blocking VGCC results in increased release of calcium from intracellular stores

**Figure 3:**
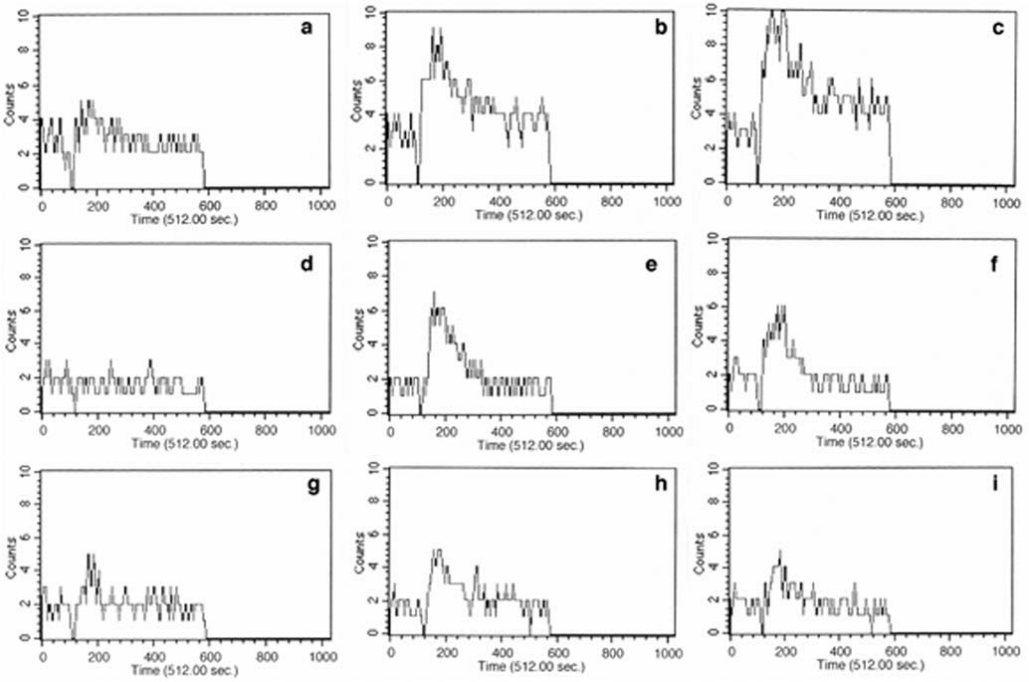
Blocking VGCC increases calcium release from intracellular stores. Real time increase in calcium influx over 5 min in CFP10-DCs stimulated with 1 MOI BCG. Prior to stimulation, DCs were blocked with anti-L-type antibody (b, e & h) or anti-R-type antibody (c, f, & i). Subpanels a, d and g represent BCG stimulation in the absence of VGCC blocking. For panels A and B, DCs were suspended in calcium sufficient (RPMI) medium and calcium (PBS) deficient medium, respectively. For panel C, DCs were incubated with 100 µM TMB-8 prior to blocking with L-type or R-type VGCC and calcium measurements were carried out in calcium sufficient (RPMI) medium. Data from one of three independent experiments are shown.

Intriguingly, since blocking calcium-inducing channels (L-type and R-type) resulted in further increasing calcium influx, it was important to identify the source of this calcium. To this end, we suspended L-type and R-type VGCC-blocked-CFP10-DCs in calcium sufficient (culture medium) or calcium deficient medium (PBS) and measured calcium influx upon BCG stimulation. As shown, under calcium sufficient conditions, both phases of calcium influx, i.e. the intracellular release followed by import from the extracellular medium (as a result of activation of CRAC channels following depletion of intracellular stores [11], [12]), could be observed (Figure 3, panels b and c). In contrast, in calcium deficient medium, i.e. in the absence of extracellular calcium, one could only observe increased release of calcium from intracellular stores (Figure 3, panels e and f). The subsequent phase of calcium influx from the extracellular medium was not observed. This indicated that blocking L-type and R-type VGCC resulted in increased release of calcium from intracellular stores followed by activation of CRAC channels that together resulted in higher mobilization of calcium in CFP10-DCs. This was further confirmed when the release from intracellular stores was inhibited using TMB-8, wherein the observed increase of both phases was blocked (Figure 3, panels h and i). One of the intracellular enzymes involved in the generation of IP3 is Phospholipase Cγ (PLCγ) [11], [12]. PLCγ acts on phosphoinositol 2 phosphate and converts it into IP3 and diacylglycerol. While IP3 binds to IP3 receptors on the endoplasmic reticulum to release calcium from intracellular stores, diacylglycerol activates protein kinase C. We therefore, specifically blocked PLCγ using a biopharmacological inhibitor and looked at calcium induction following blocking VGCC and BCG stimulation. As shown in Figure S4, inhibiting PLCγ completely blocked increase in calcium influx upon blocking VGCC (panels b and c). Put together, the above results clearly indicate the role of PLCγ in calcium induction following blocking L-type and R-type VGCC.

### Blocking L-type and R-type VGCC in DCs increases expression of Th1 promoting genes

**Figure 4:**
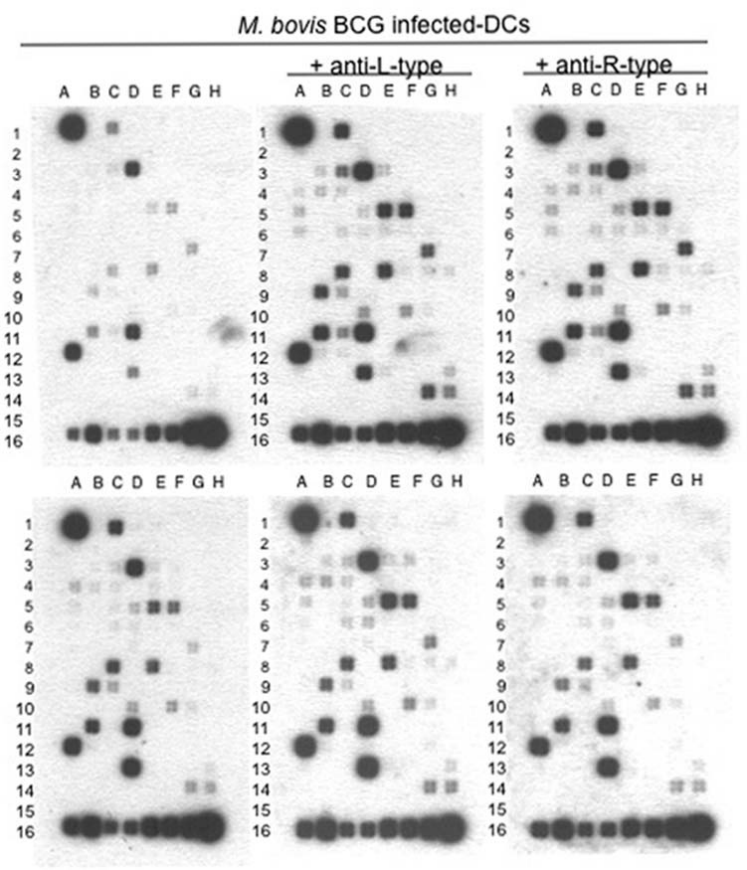
Blocking L-type and R-type VGCC in DCs induce expression of Th1 promoting genes. Expression levels of Th1/Th2/Th3 pathway specific gene array in GM-CSF-DCs (upper panels) or CFP10-DCs (lower panels) infected with 1 MOI BCG for 24 h in the presence or absence of anti-L-type or anti-R-type antibody blocking. Spot # A1, GAPDH control; spot # C15, # D15 and # E15 are negative controls. Data from one of two independent experiments are shown.

Next, we investigated modulation in the expression of genes in DCs following blocking of L-type and R-type VGCC. To this end CFP10-DCs and GM-CSF-DCs were incubated with antibodies to L-type and R-type VGCC and subsequently infected with BCG. RNA was enriched, processed and probed against a pathway specific Th1/Th2/Th3 array. As shown in Figure 4, blocking L-type and/or R-type VGCC in BCG-infected CFP10-DCs (lower panels) induced the expression of genes promoting Th1 and pro-inflammatory responses. This included Fosl1 (spot # B4), CD80 (spot B3), CD86 (spot C3), CCAAT/enhancer binding protein (C/EBP) beta (Spot # D3), Fosl2 (spot # C4), IL-12p40 (spot # F5), IL-15 (spot # C6), IL-18 (spot # D6), IL-6 (spot # G7), Jak1 (spot # E8), NF-κB1 (spot # F10), TNF (spot # D13). Increase in the levels of CD80 and CD86 upon L-type and R-type VGCC blocking indicated that these DCs would be better equipped to prime T cells. In addition message levels of IL-10 (spot # E5) were also upregulated. However, increased IL-10 protein levels were not observed in these groups (data not shown), indicating regulation at the post-transcriptional level. An essentially similar pattern was observed in BCG infected GM-CSF-DCs (upper panels) following blocking of VGCC. The expression of SOCS1 (spot # B11) and SOC3 (spot # D11) were also increased in addition to IL-10 (spot # E5). Like CFP10-DCs, BCG infected GM-CSF-DCs did not show increased IL-10 protein levels (data not shown). These results indicate that high expression of L-type and R-type VGCC in CFP10-DCs conditioned DCs to induce suppressor responses via attenuated calcium influx.

### Blocking L-type and R-type VGCC induces high IL-12 expression

Although a number of genes were upregulated following VGCC blocking, we chose to functionally validate two key genes, namely, IL-12p40 and NF-κB. This was because IL-12p40 plays a dominant role in regulating pro-inflammatory responses from DCs that are crucial for mediating protection against *M. tuberculosis* infection [6], [7]. On the other hand, NF-κB positively regulates the expression of many genes that were upregulated including TNF, IL-15 and IL-18 [27]. As shown in Figure S5, compared to GM-CSF-DCs, CFP10-DCs showed reduced activation of NF-κB as ascertained by EMSA (P<0.009). Supershift analyses showed that the bound complex consisted of c-Rel and p65 subunits (data not shown). However, blocking L-type and R-type VGCC in CFP10-DCs (and also in GM-CSF-DCs) resulted in increased activation of NF-κB. The increase in the activation was more evident with R-type blocking when compared with L-type blocking.

**Figure 5:**
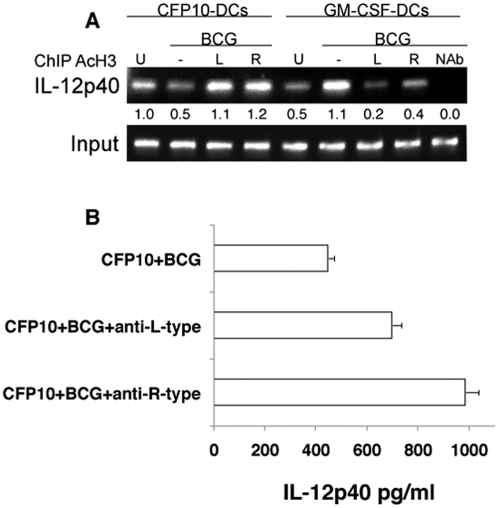
Blocking L-type and R-type VGCC induces high expression of IL-12p40. A, ChIP analysis of acetylated histone H3 (AcH3) from the IL-12p40 promoter in uninfected (U) or BCG infected CFP10-DCs or GM-CSF-DCs in the presence or absence of anti-L-type (L) or anti-R-type (R) antibody. Nab, no antibody in BCG infected GM-CSF-DCs. Data from one of three independent experiments are shown. B, IL-12p40 levels in culture supernatants of CFP10-DCs processed as in A. Data from one of three independent experiments are shown.

We next investigated whether blocking L-type and R-type VGCC results in increased expression of IL-12p40. To this end, we monitored the acetylation of histone H3 at the IL-12p40 promoter between position −121 to −131 (a region that has been functionally characterized to be important for IL-12 expression) [28], by chromatin immunoprecipitation (ChIP) and protein expression by ELISA. As shown in Figure 5A, ChIP analyses showed increased pull down of acetylated histone H3 in BCG infected GM-CSF-DCs when compared with CFP10-DCs, as evident by increased levels of the PCR amplified product. In fact, IL-12p40 levels in BCG infected CFP10-DCs were lower than uninfected DCs, thus confirming our earlier observations [23] at the transcriptional level. Specificity of ChIP was ascertained when no amplification was obtained in the group where the antibody against histone H3 was omitted. This indicated increased transcriptional activity at the IL-12p40 promoter in GM-CSF-DCs. However, blocking L-type and R-type VGCC in CFP10-DCs showed increased pull down of acetylated histone H3 resulting in increased levels of the PCR product. This indicated a direct role of blocking L-type and R-type VGCC in mediating increased IL-12p40 transcriptional activity. The reduced levels of PCR product in GM-CSF-DCs in the presence of L-type and R-type VGCC blocking could be a result of feedback regulation, since the levels were quite high upon BCG infection itself. The ChIP data corroborated very well with the protein levels of IL-12p40. Blocking L-type and R-type VGCC in BCG infected CFP10-DCs (Figure 5B) significantly increased IL-12p40 levels.

### T cells activated by VGCC-blocked-DCs mediate killing of M. tuberculosis in macrophages

**Figure 6:**
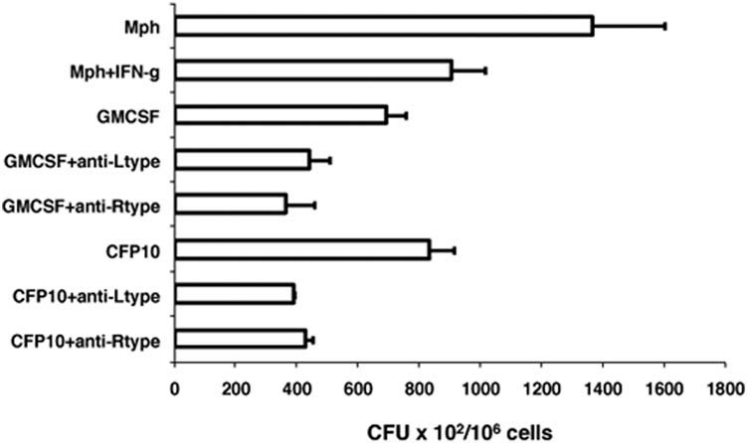
T cells activated by L-type and R-type VGCC-blocked-DCs kill *M. tuberculosis* inside macrophages. L-type or R-type VGCC-blocked-BCG-infected GM-CSF-DCs (GMCSF) or CFP10-DCs (CFP10) were co-cultured with T cells from BCG immunized mice. From the co-culture, T cells were enriched and incubated with *M. tuberculosis* H37Rv infected macrophages. Cells were lysed and CFU was determined. As controls, infected macrophages without incubation with T cells and macrophages treated with 2 ng/ml IFN-γ prior to infection with *M. tuberculosis* in the absence of T cell addition were also included. Data are the mean of three independent experiments. Error bars represent mean±s.d. P<0.01 for Mph+IFN-g vs CFP10+anti-Ltype; P<0.01 for Mph+IFN-g vs CFP10+anti-Rype and P<0.006 for Mph vs GMCSF+anti-Ltype. Two-tailed Student's t-test was employed for P values.

To investigate the functional relevance of the results obtained so far, we next tested the ability of T cells activated by VGCC-blocked DCs to kill mycobacteria inside macrophages as recently carried out with chemokine and cytokines conditioned CFP10-DCs [24]. We first ensured that blocking L-type and R-type VGCC in CFP10-DCs and GM-CSF-DCs activated T cells that secreted high levels of IFN-γ and low levels of IL-10 (data not shown). Next, BCG infected DCs were co-cultured with T cells enriched from BCG immunized mice. Form the DC-T cell co-culture, T cells were separated by MACS and incubated with *M. tuberculosis* H37Rv infected macrophages. Control groups included *M. tuberculosis* infection of resting and IFN-γ activated macrophages in the absence of any T cell addition. CFU in macrophages incubated with T cells that were activated by L-type and R-type VGCC-blocked-BCG-infected CFP10-DCs, showed a significant decrease when compared to CFU from either *M. tuberculosis* infected resting or activated macrophages or when compared with T cells activated by CFP10-DCs in the absence of VGCC blocking (Figure 6). A similar decrease in CFU were seen when T cells activated by L-type and R-type VGCC-blocked-BCG-infected GM-CSF-DCs were employed. These results indicate that blocking L-type and R-type VGCC in DCs activated T cells that subsequently mediated effective killing of virulent *M. tuberculosis* inside macrophages.

### Blocking L-type and R-type VGCC in macrophages and PBMCs kills *M. tuberculosis*

**Figure 7:**
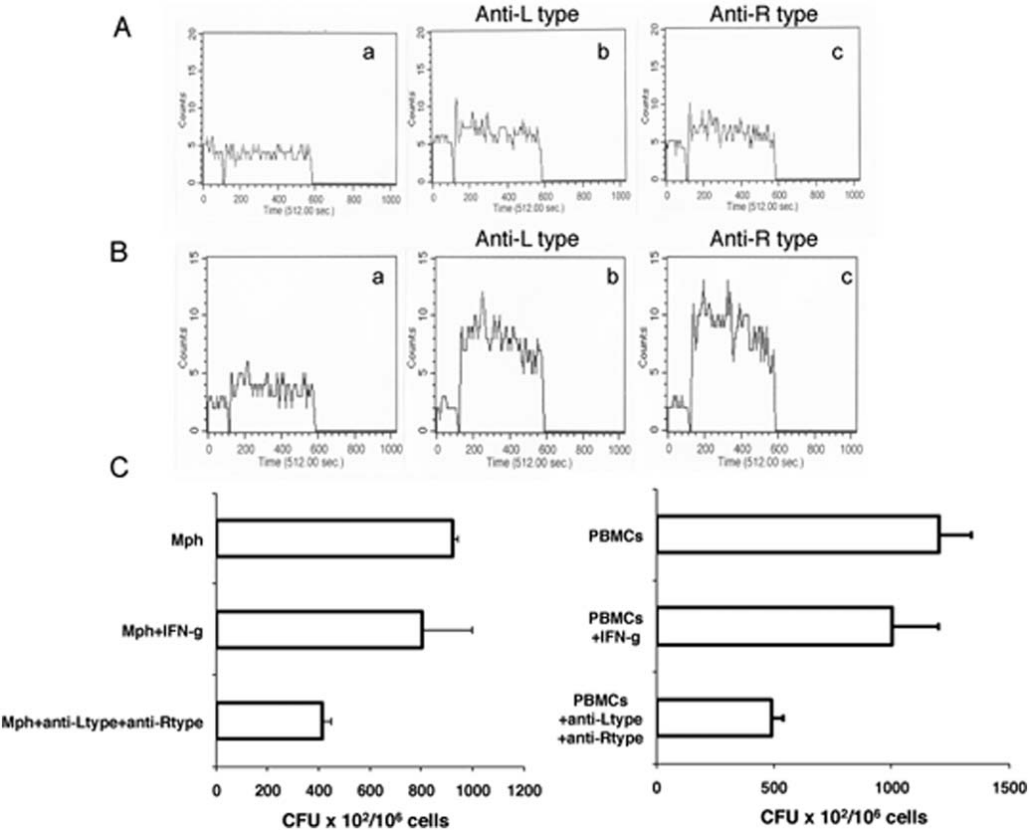
Blocking L-type and R-type VGCC in macrophages and PBMCs increases calcium influx and kills intracellular *M. tuberculosis*. Real time increase in intracellular calcium over 5 min following stimulation of mouse macrophages (A) or human PBMCs (B) with 1 MOI BCG. Subpanel a- BCG stimulation, b & c anti-L-type and anti-R-type antibody blocking prior to BCG stimulation. C, *M. tuberculosis* H37Rv CFU in macrophages (left panel) or PBMCs (right panel) in the presence or absence of anti-L-type and anti-R-type antibody. IFN-g, cells incubated with 2 ng/ml IFN-γ prior to infection with *M. tuberculosis* H37Rv. Data are the mean of three independent experiments. Error bars represent mean±s.d. In C left panel P<0.09 for Mph+IFN-g vs Mph+anti-Ltype+anti-Rtype, P<0.005 for Mph vs Mph+anti-Ltype+anti-Rtype; right panel P<0.03 for PBMCs vs PBMCs+anti-Ltype+anti-Rtype, P<0.09 for PBMCs+IFN-g vs PBMCs+anti-Ltype+anti-Rtype. Two-tailed Student's t-test was employed for P values.

Next it was relevant to investigate whether blocking L-type and R-type VGCC in mouse macrophages or human PBMCs would mediate effective killing of *M. tuberculosis*. We first monitored whether blocking L-type and R-type VGCC induced calcium influx in macrophages and PBMCs. BCG stimulation of macrophages (Figure 7A) or PBMCs (Figure 7B) did not induce appreciable levels of calcium influx (28.7 µM and 39.4 µM, respectively). However, blocking either L-type or R-type VGCC induced a significant increase in calcium mobilization in both macrophages (54.9 µM and 53.7 µM, respectively) and PBMCs (169.2 µM and 196.5 µM, respectively). This indicated that L-type and R-type VGCC played an inhibitory role in calcium mobilization even in these cells. The increase was more evident in the case of PBMCs when compared with macrophages.

Next, mouse macrophages or human PBMCs were infected with *M. tuberculosis* H37Rv for 24 h. Cells were then incubated with antibodies to L-type and R-type VGCC and cultured for 48 h. Subsequently, cells were lysed and monitored for CFU. As shown in Figure 7C, blocking L-type and R-type VGCC in either mouse macrophages (left panel) or human PBMCs (right panel) resulted in effective killing of *M. tuberculosis*. In fact the reduction was 2-fold better than that observed with IFN-γ activated macrophages or PBMCs. Similar results were obtained upon inhibition of VGCC using specific siRNAs (Figure S6). These results indicated that L-type and R-type VGCC prevent calcium responses in macrophages and PBMCs resulting in defective clearance of *M. tuberculosis*.

### PBMCs of patients with active TB disease express high levels of L-type and R-type VGCC

**Figure 8:**
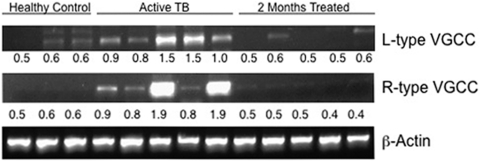
Patients with active TB disease display higher levels of L-type and R-type VGCC. RT-PCR of L-type and R-type VGCC transcripts in PBMCs from healthy controls, patients with active TB disease and the same patients following 2 months of chemotherapy. β-actin levels are depicted as loading control. For L-type P<0.053 (for healthy vs patients) and for R-type P<0.08 (for patients vs follow-ups). Two-tailed Student's t-test was employed for P values.

In order to give physiological relevance to the data obtained thus far, we looked at the levels of L-type and R-type VGCC in human cohorts of healthy controls (n = 6), patients with active TB (n = 11) and patients undergoing chemotherapy (n = 11). Five patients consented to be bled again at two months post-therapy, while six patients who had already received two months of therapy consented to give blood for the study. Figure 8 shows data from the five patients who consented to give blood at the time of diagnosis and following two months of therapy along with three healthy controls. Compared with healthy controls, PBMCs of patients with active TB disease expressed high levels of both L-type (P<0.05) and R-type (P<0.07) VGCC. Interestingly, PBMCs of the same patients following 2 months of chemotherapy, wherein their sputum became AFB^−^, now displayed highly reduced levels of L-type (P<0.05) and R-type VGCC (P<0.08). In fact the levels were very similar to or in some cases even lower than healthy controls. Similar results were obtained in the other 6 patients with active disease and patients who had taken 2 months of therapy, wherein PBMCs of patients with active TB expressed higher levels of L-type and R-type VGCC when compared with patients who had taken treatment (data not shown). These results are consistent with the data obtained in Figure 7, wherein blocking L-type and R-type VGCC in macrophages and PBMCs resulted in reduced bacterial loads.

### Blocking VGCC in vivo reduces bacterial burden in mice

**Figure 9:**
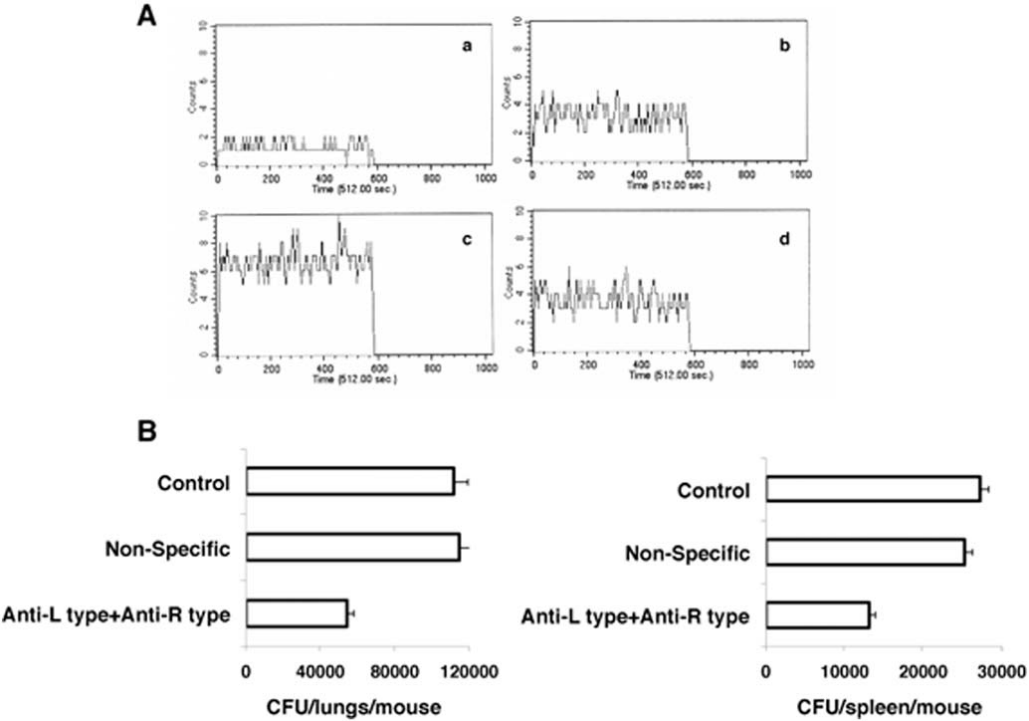
Treatment of mice with anti-L-type and anti-R-type antibodies increases intracellular calcium and reduces *M. tuberculosis* burden. A, intracellular calcium levels in splenocytes from mice infected with *M. tuberculosis* H37Rv (panel b), or mice infected with *M. tuberculosis* H37Rv followed by i.v. injection of 25 µg each of anti-L-type and anti-R-type (panel c) or non-specific antibody (panel d). Panel a shows intracellular calcium in splenocytes from naïve mice that received anti-L-type and anti-R-type antibody in the absence of *M. tuberculosis* infection. B, Mice were infected i.v. with *M. tuberculosis* H37Rv followed by i.v. injection of 25 µg each of anti-L-type and anti-R-type or non-specific antibody 7 d later. Mice were sacrificed and lungs and spleen homogenates were plated for determining CFU after 7 d following antibody treatment. Control represents *M. tuberculosis* H37Rv infected mice treated with PBS. Data are the mean of three independent experiments. Error bars represent mean±s.d. P<0.001 in lungs for control vs anti-L type+anti-R type, P<0.006 in lungs for non-specific antibody vs anti-L type+anti-R type, P<0.003 in spleen for control vs anti-Ltype+anti-Rtype, P<0.005 in spleen for non-specific antibody vs anti-L type+anti-R type. Two-tailed Student's t-test was employed for P values.

In order to test proof of principle, we next investigated the role of L-type and R-type VGCC in regulating *M. tuberculosis* infection in mice. To this end, we first infected mice with *M. tuberculosis* H37Rv. Post-exposure we followed it up by injection of antibodies to VGCC. We first investigated whether antibody treatment would indeed increase intracellular calcium in vivo. As shown in Figure 9A, splenocytes from *M. tuberculosis* infected mice showed higher calcium levels when compared to splenocytes from uninfected mice. However, following anti-L-type and anti-R-type VGCC antibody injection in infected mice, intracellular calcium levels in splenocytes were significantly higher when compared either in the absence of antibody treatment or following injection of a non-specific antibody. This indicated that injection of antibodies to VGCC elevates intracellular calcium concentrations in cells in vivo during *M. tuberculosis* infection. In the next experiment, mice were infected with *M. tuberculosis* H37Rv and seven days later mice were injected with antibodies to L-type and R-type VGCC and bacterial loads in lungs and spleen were determined seven days following injection of antibodies. As shown in Figure 9B, CFU counts in lungs were reduced by 50% in mice that received antibodies to L-type and R-type VGCC when compared with mice that received a non-specific antibody (P<0.006) or control (P<0.001). Similarly, the CFU in spleen were also reduced by 50% upon injection of VGCC specific antibodies when compared with mice that received non-specific antibody (P<0.005). These results indicate that blocking VGCC in vivo results in reduction of *M. tuberculosis* infection as a result of increased calcium influx in infected cells.

## Discussion

*M. tuberculosis* has devised numerous ways to evade protective immune responses [3]–[5] by modulation of host factors. These include downmodulation of surface MHC class II expression on macrophages and changes in the profile of cytokines and chemokines in DCs that individually and collectively affect priming of T cells [5]. Other modulations include downregulation of reactive nitrogen and reactive oxygen species generation, inhibition of IFN-γ receptor expression and activation on macrophages [29], [30]. A number of immune evasive effects of *M. tuberculosis* are reproduced by specific *M. tuberculosis* antigens. These include inhibition of IFN-γ and Toll Like Receptor pathways by 19 kDa lipoprotein [31], inhibition of NF-κB activation and IL-12 production by mannosylated lipoarabinomanan mediated activation of DC-SIGN [32]. On similar lines, we have also demonstrated the immuno-suppressive effects of CFP-10 that mediates its effects by inducing differentiation of DCs that confer suppressor responses [21], [24].

Calcium plays a key role in regulating many of the modulations that are targeted by *M. tuberculosis* for immune evasion. For example, calcium regulates the activities of calmodulin and sphingosine kinase that affects *M. tuberculosis* survival [16]; and calcinuerin that regulates the expression of coronin-1 on phagosomes thus affecting phagosome maturation [33]. In addition, calcium concentrations and kinetics directly affect the activation of transcription factors such as NF-AT and NF-κB leading to differential cytokine expression and this governs the quantum and quality of immune responses [34]. As described earlier, calcium influx in mammalian cells is stage dependent, initiating with the depletion of intracellular stores followed by opening up of various channels [10], [11]. Although, VGCC have been shown to play major roles in physiological responses, their roles in infectious diseases have recently assumed importance. For example, L-type VGCC has been demonstrated to play roles in regulating intracellular growth of *Legionella pneumophila* [19] and in induction of calcium in CD4^+^ T cells during *Leishmania* infection [35].

Therefore, in the present study, we investigated the roles of L-type and R-type VGCC in mediating protective responses during *M. tuberculosis* infection. Our results showed that inhibiting or blocking L-type and R-type VGCC in DCs, macrophages and human PBMCs increased intracellular calcium concentration (blocking PQ-type and T-type VGCC completely inhibited even basal calcium induction and hence were not pursued further; data not shown). This indicated a negative role for L-type and R-type VGCC in calcium influx in the context of *M. tuberculosis* infection. L-type VGCC has been previously reported to play a negative role in calcium induction wherein the knockdown of its beta subunit in mouse results in increased frequency of calcium oscillations leading to increased insulin secretion in beta cells [36]. In addition, blocking L-type and R-type VGCC increased the expression and kinetics of a number of cytokines (in particular IL-12) and transcription factors that promote pro-inflammatory responses. Several reports indicate a role for VGCC in regulating the activation of transcription factors. For example, L-type VGCC has been shown to be essential for CREB phosphorylation [37] and activation of *c-fos* [38].

We next showed that L-type and R-type VGCC directly affected survival of *M. tuberculosis*. Blocking L-type and R-type VGCC in macrophages or PBMCs resulted in killing of *M. tuberculosis*. Blocking L-type and R-type VGCC in DCs, primed T cells that mediated killing of *M. tuberculosis* inside macrophages. The fact that *M. tuberculosis* killing was better than that obtained by IFN-γ treatment indicates that blocking L-type and R-type VGCC could bypass the suppressive effects of *M. tuberculosis* on IFN-γ mediated clearance. Blocking VGCC in infected macrophages also induced increased cleavage of caspase-3 (Figure S7). It has been shown that *M. tuberculosis* prevents apoptosis of infected macrophages leading to decreased antigen presentation [5]. Calcium also induces caspase-3 activation [39], thereby indicating a role for L-type and R-type VGCC blocking in calcium mediated caspase activation during *M. tuberculosis* infection. *M. tuberculosis* has been shown to interact differently with DCs and macrophages. These include opposite effects on MHC class II levels, IL-12 and IFN-γ secretion and regulation [30], [31], [40]. In this study, we have identified a common factor in the form of L-type and R-type VGCC that negatively governs protective responses from both DCs and macrophages that could be targeted for therapeutic intervention.

It is pertinent to mention here that the role of VGCC in DCs has been a subject of contention. While some report the presence of active VGCC in DCs [41], others observed that calcium influx is mainly via CRAC channels [42]. Our data indicate that these channels play a direct role in generation of immune responses from DCs and macrophages. The role of L-type VGCC in CD4^+^ T cells has recently been shown in the context of *Leishmania* infection wherein despite being non-excitable, these T cells express functional L-type VGCC [35]. VGCC in these T cells play a major role in inducing calcium influx with their association with the scaffold protein AHNAK-1 [35]. Therefore, the data on T cells add support to our results, wherein these channels directly influence functional outcomes in non-excitable cells.

The negative role of L-type and R-type VGCC during *M. tuberculosis* infection was further established with our in vivo data, wherein blocking VGCC in *M. tuberculosis* infected mice significantly reduced bacterial loads in infected mice. The in vivo data correlated well with our results in human cohorts, wherein high expression of L-type and R-type VGCC was observed in patients with active TB disease when compared with healthy controls. Following chemotherapy, the levels of these VGCC decreased significantly. Furthermore, blocking VGCC in PBMCs of healthy or TB patient increased the expression levels of granulysin, IFN-γ receptor2 that are known to mediate killing of *M. tuberculosis* and also downregulated the expression of genes such as CCL2 that promotes Th2 responses (data not shown) pointing to possible downstream mechanisms that would together bring about a reduction in *M. tuberculosis* burden in infected cells. Interestingly, blocking these VGCC inhibited invasion of erythrocytes by *Plasmodium falciparum* and this indicated that these channels play a role during infections by other pathogens (data not shown).

Collectively, our results suggest that L-type and R-type VGCC play important roles in regulating immune responses during *M. tuberculosis* infection. Inhibition of these channels results in significant increase in calcium mobilization leading to expression of pro-inflammatory genes and the generation of protective immunity to mycobacteria. Significantly, our results on patient samples further indicate that these channels are expressed at high levels during active disease, indicating a negative role played by these VGCC during *M. tuberculosis* infection. Finally, the reduction of *M. tuberculosis* infection in mice treated with antibodies to L-type and R-type VGCC indicates their potent roles in determining the course of infection during different stages of *M. tuberculosis* infection and TB disease.

## Materials and Methods

### Animals

Female BALB/c mice 4–6 wk of age kept in pathogen free environment and all experiments were conduced following approval from the ICGEB animal ethics committee.

### Human Studies

All experiments were conducted following approval by the human ethics committee of LRS Institute of TB & Respiratory diseases. Following written informed consent venous blood from PPD^+^ healthy volunteers or patients in the age group of 15–60 years freshly diagnosed with active TB (based on acid fast bacilli (AFB)^+^ sputum smear, chest X-Ray and clinical examination) was drawn and PBMCs were enriched. None of the healthy volunteers or patients had any previous record of TB or anti-tuberculosis treatment. Further, either HIV^+^ or immuno-compromised or pregnant women were excluded from the study.

### Materials

Antibodies to L-type Ca^2+^ α1C (cat # sc-25686) and R-type Ca^2+^ α1E (cat # sc-16225) VGCC, NF-κB p65 subunit (cat # sc-7151), siRNA against L-type Ca^2+^ CP α1C (cat # sc-42689) and R-type Ca^2+^ CP α1E VGCC (cat # 42703) and NF-κB binding consensus oligonucleotides were purchased from Santa-Cruz Biotechnologies. ELISA kits for cytokines were purchased from R&D systems. Acetylated Histone 3 ChIP kits were from Upstate Biotechnology, Inc. Lake Placid, NY. Defined pathway specific mouse Th1/Th2/Th3 (cat # OMM-034) along with True labeling AMP™ 2.0 kits (cat # GA030) were from SuperArray.

### Expression and purification of CFP-10

Endotoxin free CFP-10 was recombinantly expressed and purified as a His-tagged protein from *E. coli* as described earlier [21]–[24]. The endotoxin levels were estimated to be 0.3 EU per mg protein.

### Enrichment of DC precursors from bone marrow and generation of DCs

DCs were differentiated with either GM-CSF or CFP-10 as described before [21]–[24]. Briefly, bone marrow from the tibias and femurs of BALB/c mice were flushed out and lymphocytes and I–A^+^ cells were depleted following MACS. Cells were cultured in RPMI 1640 medium containing 10% FCS, 0.05 M 2-mercaptoethanol, 1 mM sodium pyruvate plus either 15 ng/ml GM-CSF or 20 µg/ml CFP-10. DCs differentiated with CFP-10 are referred as CFP10-DCs, while DCs differentiated with GM-CSF are referred as GM-CSF-DCs. We have shown that this method gives a homogenous population that is 99% DCs with negligible contaminating monocytes or macrophages [21], [23].

### Enrichment of macrophages and PBMCs

Mouse peritoneal macrophages were flushed using RPMI 1640 medium using a sterile syringe and an 18 guage needle. The cells were washed in PBS. Human PBMCs were enriched from heparinized blood by Ficoll-Histopaque density gradient centrifugation. Cells at the interface were removed and washed in PBS. Enriched macrophages and PBMCs were cultured in RPMI 1640 medium containing 10% FCS, 0.05 M 2-mercaptoethanol, 1 mM sodium pyruvate.

### Infection of cells with M. bovis BCG and *M. tuberculosis* H37Rv

*M. bovis* BCG and *M. tuberculosis* H37Rv were grown in Middlebrook 7H9 liquid medium supplemented with albumin/dextrose/catalase (ADC) at a final concentration of 5 g/l, 2 g/l and 0.003 g/l, respectively, along with 0.05% Tween 80. Aliquots were frozen at −85°C and viable bacteria were enumerated by plating serial dilutions on 7H11 agar. DCs were infected with BCG, while mouse peritoneal macrophages and human PBMCs were infected with *M. tuberculosis* H37Rv at 1 MOI for different times. Cells were processed either for measuring intracellular calcium influx or co-cultured with T cells or for monitoring colony forming unit (CFU) as described below.

### Binding of antibodies to L-type and R-type VGCC to DCs

Antibodies to L-type Ca^2+^ α1C and R-type Ca^2+^ α1E VGCC and NF-κB p65 subunit were biotinylated using NHS biotin as per standard protocols. DCs were incubated with Fc-block (BD Biosciences) followed by incubation with the above antibodies at 1 µg/10^6^ cells at 4°C for 30 min. Cells were washed and counter stained with streptavidin-PE. FACS was performed using FACS Calibur (Beckton & Dickinson) and the data were analyzed employing the CellQuest Pro software.

### Estimation of intracellular calcium levels

Intracellular calcium levels were monitored essentially as described before [24]. Briefly, either 2×10^7^/ml GM-CSF-DCs or CFP10-DCs or mouse macrophages or human PBMCs were loaded with 1 µM FLUO-3-AM for 45 min at 37°C in culture medium. The cells were thoroughly washed with HBSS and suspended in fresh culture medium. An aliquot of cells was diluted in culture medium and when required stimulated with 1 MOI BCG and real time increase in intracellular calcium concentration was monitored immediately over a period of 5 min by FACS using FACS Calibur (Beckton & Dickinson) and the data were analyzed employing the CellQuest Pro software. For some groups, cells were incubated with 2 µg/ml of L-type or R-type VGCC for 30 min. Alternatively, DCs transfected with siRNA against L-type or R-type VGCC were used for measuring calcium influx as described below. For CFP10-DCs and GM-CSF-DCs the voltage gains were adjusted so as to obtain a similar baseline prior to stimulation. The gates selected to plot the histograms within CFP10-DCs and GM-CSF-DCs with different treatments are the same.For some experiments, mice (4 mice/group) were infected intravenously with 1×10^6^ *M. tuberculosis* H37Rv for 24 h followed by intravenous injection of 25 µg each of anti-L-type and anti-R-type or a non-specific antibody. Splenocytes were excised 4 h later and loaded with 1 µM FLUO-3-AM for 45 min in culture medium. Intracellular calcium levels were measured by acquiring cells for a period of 5 min by FACS as mentioned above. No stimulations were made during the acquisition. A control group wherein naïve mice were injected with 25 µg each of anti-L-type and anti-R-type was also included to see any effect of the antibody in the absence of *M. tuberculosis* infection.

### Transfection of DCs with siRNA

5×10^6^/ml bone marrow precursors were transfected with 60 pmoles of siRNA against L-type and R-type VGCC for 72 h using the Hiperfect transfection reagent (Qiagen) in OPTIMEM medium (Invitrogen). 5 h following transfection either GM-CSF or CFP-10 was added and the incubation continued for 72 h for DC differentiation. Subsequently, RNA was enriched using TRIZOL reagent and levels of VGCC were monitored by RT-PCR. Alternatively, cells were processed for calcium measurements as described above.

### Time-lapse confocal video microscopy

1×10^5^ DCs were seeded in RPMI 1640 culture medium supplemented with 10% FCS in 30 mm glass-bottom micro well dishes in 150 µl for 14–16 h. DCs were incubated with blocking antibodies to L-type and R-type VGCC for 1 h and then loaded with Fluo-3-AM for 45 min at 37°C. The cells were washed two times with phenol red free RPMI-1640 medium. Confocal live cell imaging was performed with Nikon TE-2000-E laser-scanning confocal microscope (Nikon, Japan) with 60× objective magnification, numerical aperture 1.4, PlanApo optics, equipped with Argon laser, using excitation and emission wavelength of 488 and 516, respectively. The images were acquired with a frame rate of 2 seconds for a total duration of 180 s and 90 frames were recorded. The cells were stimulated with 10 µg/ml *M. tuberculosis* whole cell lysate (WCL) at the 15^th^ frame (30 s).

### Quantitative and semi-quantitative RT-PCR

Total RNA from cells processed differently was isolated. RNA was employed in real time quantitative RT-PCR using SYBRgreen on a Bio-Rad iCycler. The expression level of a gene in a given sample was represented as 2^−ΔΔCT^, where ΔΔCT = [ΔCT_(sample, infected)_] – [ΔCT_(sample, uninfected)_] and ΔCT = [CT_(sample)_] – [CT_(β-actin)_], where β-actin is the housekeeping gene. Semi-quantitative RT-PCR was carried out on a Bio-RAD MyCycler. The following primers were used: for mouse L-type VGCC forward 5′ GGCTGGAGGTGACATCGAGGG 3′ and reverse 5′ GAGGCAATGGAGCGCACTGAG 3′ at 95°C 1 min, 54°C 1 min, 72°C 1 min; for mouse R-type VGCC forward 5′ TCGACAGTGGTGAACATTAGC 3′ and reverse 5′ CGCTTGATGGTTTTCAGTGGC 3′ at 95°C 1 min, 55°C 1 min, 72°C 1 min; for human L-type VGCC forward 5′ AGTCCGTCAACACCGAAAAC 3′ and reverse 5′ CCAGTTGGGCTGGTTGTAGT 3′ at 95°C 1 min, 56°C 1 min, 72°C; for human R-type VGCC forward 5′ ATGACGGTCCACTTCACCTC 3′ and reverse 5′ AGAGACTGCCGTTCTTGGAA 3′ at 95°C 1 min, 60°C 1 min, 72°C; mouse β-actin forward 5′ TGTTACCAACTGGGACGACA 3′ and reverse 5′ AAGGAAGGCTGGAAAAGAGC 3′ at 95°C 1 min, 60°C 1 min, 72°C 1 min; and human β-actin forward 5′ AGAAAATCTGGCACCACACC 3′ and reverse 5′ AGGAAGGAAGGCTGGAAGAG 3′ at 95°C 1 min, 60°C 1 min, 72°C 1 min. The products were separated on 1% agarose gel and visualized.

### Microarray analyses

All steps were conducted strictly following the manufacturer's (SuperArray) protocol. DCs were infected with BCG for 24 h in the presence and absence of blocking antibodies to L-type and R-type VGCC. Total RNA was enriched and 2 µg RNA was processed and converted into c-RNA. Following normalization c-RNA was probed against pathway specific Th1/Th2/Th3 oligo-GEArrays.

### Elecrophoretic Mobility Shift Assays (EMSA)

DCs were infected with 1 MOI BCG for indicated times and nuclear extracts were prepared as described elsewhere [24]. Briefly, at the end of the incubation cells were chilled on ice and washed once with ice-cold PBS and lysed in buffer containing 10 mM HEPES (pH 7.9); 10 mM KCl; 0.1 mM EDTA; 0.1 mM EGTA, 0.5% Nonidet P-40, and 2 µg/ml each of aprotinin, leupeptin and pepstatin. The suspension was centrifuged at 13,000 rpm for 1 min at 4°C. The nuclear pellet was resuspended in ice-cold extraction buffer- 20 mM HEPES, pH 7.9; 0.4 M NaCl; 1 mM EDTA; 1 mM EGTA; 1 mM DTT; 1 mM PMSF and 2 µg/ml each of aprotinin, leupeptin and pepstatin. EMSA were performed by incubating 12–15 µg of nuclear extract with ^32^P-end-labeled 19-mer double stranded consensus NF-κB oligonucleotide sequence for 15 min at 37°C. The incubation mixture included 2–3 µg of poly(dI.dC) in a binding buffer (25 mM Hepes, pH 7.9; 0.5 mM EDTA; 0.5 mM DTT; 1% Nonidet P-40; 5% glycerol; 50 mM NaCl). The DNA-protein complex formed was separated from free oligonucleotide on 5% native polyacrylamide gel using buffer containing 50 mM Tris, 200 mM glycine (pH 8.5), and 1 mM EDTA, and the gel was then dried. The specificity and extent of binding was examined by competition with unlabeled (cold) oligonucleotide. For supershift assays, nuclear extracts were incubated with antibodies against either c-Rel or p65 subunits of NF-κB for 30 min at room temperature before the complex was analyzed by EMSA.

### Chromatin Immunoprecipitation (ChIP) Assay

The ChIP procedure was carried out following the manufacturer's instructions (Upstate Biotechnology) with some modifications. Briefly, following specific stimulus, 3×10^6^ cells were fixed with 1% formaldehyde for 10 min at 37°C and quenched with 0.125 M glycine for 10 min at room temperature. Chromatin was sheared to an average size of <500 bp and precleared with protein A-agarose beads. The soluble chromatin was incubated overnight with 2–3 µg of anti-acetylated histone H3 followed by incubation with the blocked beads. The immune complexes were collected by centrifugation and washed following the manufacturer's protocol. Input and immunoprecipitated chromatin samples were reverse cross-linked by incubation at 65°C overnight in presence of 200 nM NaCl. Following proteinase K digestion, DNA was extracted with phenol/chloroform and precipitated with ethanol. Precipitated DNA was diluted serially, analyzed by PCR consisting of 30 amplification cycles, and resolved on agarose gel.

### Enrichment of T cells

BALB/c mice were immunized with 1×10^6^ BCG subcutaneously at base of tail. Seven days later, inguinal lymph nodes were excised. From this B cells, macrophages and DCs were removed following MACS using anti-B220^+^, anti-CD11c^+^ and anti-CD11b^+^ microbeads. The negatively selected T cells were 98% pure as determined by CD90-PE staining. The percentage of I–A^+^ cells in T cell preparations was 0.5%.

### Intracellular survival of mycobacteria

DCs were infected with 1 MOI BCG for 24 h in the presence and absence of antibodies to L-type and R-type VGCC as described above. DCs were then co-cultured for 48 h with BCG-specific T cells enriched from immunized mice. From this co-culture DCs were selectively depleted and T cells were cultured for 48 h with *M. tuberculosis* H37Rv infected macrophages. Cells were lysed and plated in serial dilutions onto 7H11 agar plates. Alternatively, mouse peritoneal macrophages or human PBMCs were infected with 1 MOI *M. tuberculosis* H37Rv for 24 h. Infected cells were then washed and incubated with antibodies to L-type and R-type VGCC for a further 48 h. Cells were lysed and plated in serial dilutions onto 7H11 agar plates. Two to three week later plates were scored for Colony Forming Units (CFU).

### Infection of mice with *M. tuberculosis*

Groups of naïve mice (4/group) were infected with 1×10^6^ *M. tuberculosis* H37Rv via the tail vein. One group of mice was sacrificed 24 h later and lung homogenates were plated onto 7H11 agar plates for confirming infection. Seven days post infection, 25 µg each of anti-L-type and anti-R-type or a non-specific antibody was injected into the tail vein of mice. Seven days following injection, mice were sacrificed and lung and spleen cells were enriched using a homogenizer. An aliquot of the homogenate was lysed and plated onto 7H11 agar plates in serial dilutions for CFU monitoring.

### Statistics

Two-tailed Students t test was used to compare the statistical significance.

## Supporting Information

Figure S1 Antibodies to L-type and R-type VGCC bind DCs. Antibodies to L-type Ca2+ α1C (cat # sc-25686) and R-type Ca2+ α1E (cat # sc-16225) VGCC and NF-κB p65 subunit (cat # sc-7151) were biotinylated using NHS biotin as per standard protocols. Cells were washed and counter stained with streptavidin-PE. FACS was performed using FACSCalibur (Beckton & Dickinson) and the data were analyzed employing the CellQuest Pro software. Histograms depict surface levels of L-type (a & d) and R-type (b & e) VGCC on CFP10-DCs (a–c) and GM-CSF-DCs (d–f). Histograms (c & f) depict binding of anti-NF-κB p65 (used as non-specific control). The thin lines depict staining with streptavidin-PE, while the thick lines depict staining with specific antibody. One of three independent experiments is shown.
(0.10 MB TIF)

Figure S2 siRNA mediated silencing of L-type and R-type VGCC. 5×106/ml bone marrow precursors were transfected with 60 pmoles of siRNA against L-type and R-type VGCC for 72 h using the Hiperfect transfection reagent (Qiagen) in OPTIMEM medium (Invitrogen). 5 h following transfection either CFP-10 (Panel A) or GM-CSF (Panel B) was added and the incubation continued for 72 h for DC differentiation. Subsequently, RNA was enriched using TRIZOL reagent and levels of VGCC were monitored by RT-PCR. C, control untransfected DCs. NS, DCs transfected with siRNA against- firefly luciferase (used as non-specific control). L, DCs transfected with siRNA against L-type VGCC. R, DCs transfected with siRNA against R-type VGCC. Lower panel represents β-actin as loading controls.
(0.09 MB TIF)

Figure S3 Blocking L-type and R-type VGCC in CFP10-DCs increases calcium upon M. tb whole cell lysate stimulation. Increase in intracellular calcium levels in CFP10-DCs upon 10 µg/ml M. tb whole cell lysate stimulation measured by live cell imaging using time-lapse video confocal microscopy is shown. DCs were stimulated at frame # 15 and data on a total of 90 frames were collected and analyzed using the Image-Pro AMS6.0 software. The values were normalized to unity in order to represent all groups in a single graph. CFP10-DCs (Blue), CFP10-DCs+L-type VGCC blocking (Red), CFP10-DCs+R-type VGCC blocking (Green). Data are representative of three independent experiments.
(0.10 MB TIF)

Figure S4 Inhibiting PLCγ inhibits calcium induction following blocking of VGCC. Real time increase in calcium influx over 5 min in CFP10-DCs stimulated with 1 MOI BCG. Prior to stimulation, DCs were incubated with specific PLCγ inhibitor U73122 for 30 min followed by incubation with antibodies to L-type and R-type antibody. Panel a, CFP10-DCs treated with U73122, panel b and c, U73122 treated CFP10-DCs incubated with anti-L-type and anti-R-type antibodies, respectively.
(0.07 MB TIF)

Figure S5 Blocking L-type and R-type VGCC induces increased activation of NF-κB. A, GM-CSF-DCs or CFP10-DCs were infected with 1 MOI BCG for indicated times. EMSA for NF-κB was carried out with 10–14 µg of nuclear extracts. Arrow points to the specific band. GM-CSF-DCs (B) or CFP10-DCs (C) DCs were incubated with blocking antibody to L-type or R-type VGCC prior to infection with BCG. Data are representative of three to five independent experiments.
(0.13 MB TIF)

Figure S6 Inhibiting L-type and R-type VGCC using siRNA in macrophages and PBMCs kills intracellular M. tb. Mouse macrophages (Mph) (upper panel) or human PBMCs (lower panel) were transfected with siRNAs against L-type and R-type followed by infection with M. tb H37Rv. 48 h post-infection cell lysates were plated for CFU monitoring. IFN-g, cells incubated with 2 ng/ml IFN-γ prior to infection with M. tb H37Rv. Data are the mean of two independent experiments. Error bars represent mean±s.d. For macrophages P<0.007 for Mph vs Mph+anti-Ltype+anti-Rtype. P<0.01 for PBMCs vs PBMCs+anti-Ltype+anti-Rtype, P<0.03 for PBMCs+IFN-g vs PBMCs+anti-Ltype+anti-Rtype. Two-tailed Student's t-test was employed for P values.
(0.07 MB TIF)

Figure S7 Blocking L-type and R-type VGCC in M. tb infected macrophages induces caspase 3 activation. Western blots of cleaved and total caspase 3 levels in mouse peritoneal macrophages infected with 1 MOI M. tb H37Rv for 24 h. In some groups L-type and R-type VGCC were blocked with antibody and the incubation continued for 24 h. Data are representative of two independent experiments.
(0.06 MB TIF)

Movie S1 CFP10-DCs induce weak calcium influx. CFP10-DCs were loaded with FLUO-3-AM. Following acquisition of 15 frames as baseline, DCs were stimulated with 10 µg/ml M. tb whole cell lysate. A total of 90 frames were recorded. The movie depicts frames # 7–55. Each scanning frame has been taken at an interval of 2 seconds.
(9.64 MB AVI)

Movie S2 Blocking L-type VGCC in CFP10-DCs induces strong influx of calcium. CFP10-DCs were incubated with antibody to L-type VGCC and subsequently loaded with FLUO-3-AM. Following acquisition of 15 frames as baseline, DCs were stimulated with 10 µg/ml M. tb whole cell lysate. A total of 90 frames were recorded. The movie depicts frames # 7–55. Each scanning frame has been taken at an interval of 2 seconds.
(9.64 MB AVI)

Movie S3 Blocking R-type VGCC in CFP10-DCs induces strong influx of calcium. CFP10-DCs were incubated with antibody to R-type VGCC and subsequently loaded with FLUO-3-AM. Following acquisition of 15 frames as baseline, DCs were stimulated with 10 µg/ml M. tb whole cell lysate. A total of 90 frames were recorded. The movie depicts frames # 7–55. Each scanning frame has been taken at an interval of 2 seconds.
(9.64 MB AVI)
