# Supplementary figures and images for: Voltage Gated Calcium Channels Negatively Regulate Protective Immunity to Mycobacterium tuberculosis
Source: PLoS One. 2009 Apr 23;4(4):e5305. doi: 10.1371/journal.pone.0005305 (PMC2669286; doi:10.1371/journal.pone.0005305)

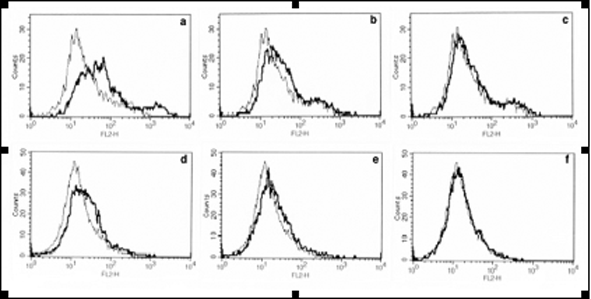

Supplement: Figure S1 — Antibodies to L-type and R-type VGCC bind DCs. Antibodies to L-type Ca2+ α1C (cat # sc-25686) and R-type Ca2+ α1E (cat # sc-16225) VGCC and NF-κB p65 subunit (cat # sc-7151) were biotinylated using NHS biotin as per standard protocols. Cells were washed and counter stained with streptavidin-PE. FACS was performed using FACSCalibur (Beckton & Dickinson) and the data were analyzed employing the CellQuest Pro software. Histograms depict surface levels of L-type (a & d) and R-type (b & e) VGCC on CFP10-DCs (a–c) and GM-CSF-DCs (d–f). Histograms (c & f) depict binding of anti-NF-κB p65 (used as non-specific control). The thin lines depict staining with streptavidin-PE, while the thick lines depict staining with specific antibody. One of three independent experiments is shown. (0.10 MB TIF) [file pone.0005305.s001.tif]

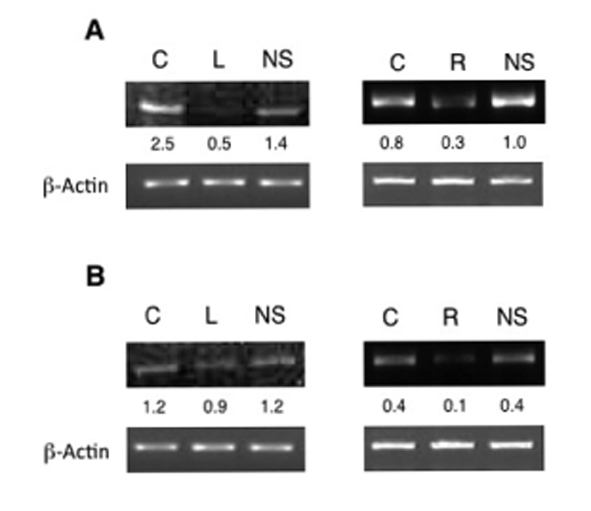

Supplement: Figure S2 — siRNA mediated silencing of L-type and R-type VGCC. 5×10^6^/ml bone marrow precursors were transfected with 60 pmoles of siRNA against L-type and R-type VGCC for 72 h using the Hiperfect transfection reagent (Qiagen) in OPTIMEM medium (Invitrogen). 5 h following transfection either CFP-10 (Panel A) or GM-CSF (Panel B) was added and the incubation continued for 72 h for DC differentiation. Subsequently, RNA was enriched using TRIZOL reagent and levels of VGCC were monitored by RT-PCR. C, control untransfected DCs. NS, DCs transfected with siRNA against- firefly luciferase (used as non-specific control). L, DCs transfected with siRNA against L-type VGCC. R, DCs transfected with siRNA against R-type VGCC. Lower panel represents β-actin as loading controls. (0.09 MB TIF) [file pone.0005305.s002.tif]

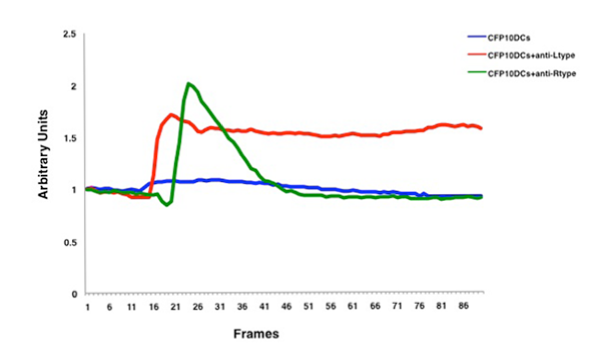

Supplement: Figure S3 — Blocking L-type and R-type VGCC in CFP10-DCs increases calcium upon M. tb whole cell lysate stimulation. Increase in intracellular calcium levels in CFP10-DCs upon 10 µg/ml M. tb whole cell lysate stimulation measured by live cell imaging using time-lapse video confocal microscopy is shown. DCs were stimulated at frame # 15 and data on a total of 90 frames were collected and analyzed using the Image-Pro AMS6.0 software. The values were normalized to unity in order to represent all groups in a single graph. CFP10-DCs (Blue), CFP10-DCs+L-type VGCC blocking (Red), CFP10-DCs+R-type VGCC blocking (Green). Data are representative of three independent experiments. (0.10 MB TIF) [file pone.0005305.s003.tif]

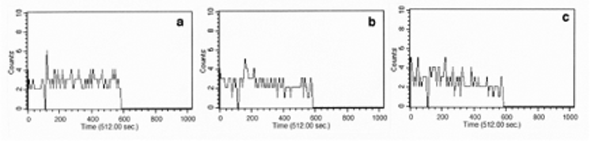

Supplement: Figure S4 — Inhibiting PLCγ inhibits calcium induction following blocking of VGCC. Real time increase in calcium influx over 5 min in CFP10-DCs stimulated with 1 MOI BCG. Prior to stimulation, DCs were incubated with specific PLCγ inhibitor U73122 for 30 min followed by incubation with antibodies to L-type and R-type antibody. Panel a, CFP10-DCs treated with U73122, panel b and c, U73122 treated CFP10-DCs incubated with anti-L-type and anti-R-type antibodies, respectively. (0.07 MB TIF) [file pone.0005305.s004.tif]

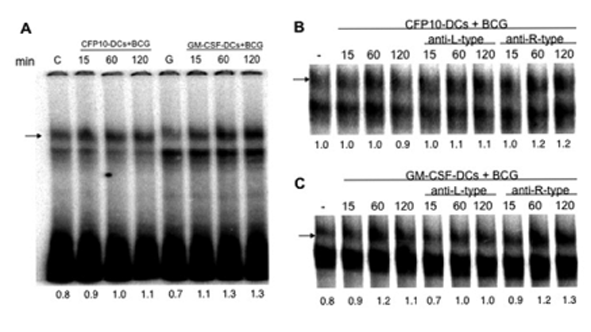

Supplement: Figure S5 — Blocking L-type and R-type VGCC induces increased activation of NF-κB. A, GM-CSF-DCs or CFP10-DCs were infected with 1 MOI BCG for indicated times. EMSA for NF-κB was carried out with 10–14 µg of nuclear extracts. Arrow points to the specific band. GM-CSF-DCs (B) or CFP10-DCs (C) DCs were incubated with blocking antibody to L-type or R-type VGCC prior to infection with BCG. Data are representative of three to five independent experiments. (0.13 MB TIF) [file pone.0005305.s005.tif]

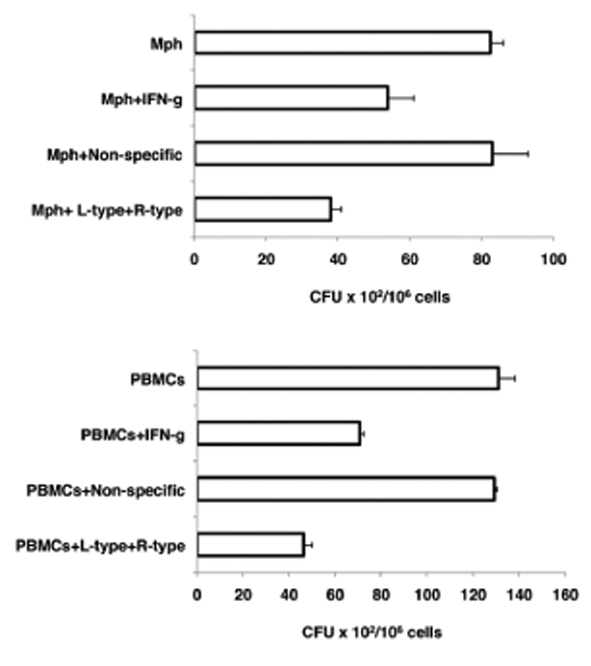

Supplement: Figure S6 — Inhibiting L-type and R-type VGCC using siRNA in macrophages and PBMCs kills intracellular M. tb. Mouse macrophages (Mph) (upper panel) or human PBMCs (lower panel) were transfected with siRNAs against L-type and R-type followed by infection with M. tb H37Rv. 48 h post-infection cell lysates were plated for CFU monitoring. IFN-g, cells incubated with 2 ng/ml IFN-γ prior to infection with M. tb H37Rv. Data are the mean of two independent experiments. Error bars represent mean±s.d. For macrophages P<0.007 for Mph vs Mph+anti-Ltype+anti-Rtype. P<0.01 for PBMCs vs PBMCs+anti-Ltype+anti-Rtype, P<0.03 for PBMCs+IFN-g vs PBMCs+anti-Ltype+anti-Rtype. Two-tailed Student's t-test was employed for P values. (0.07 MB TIF) [file pone.0005305.s006.tif]

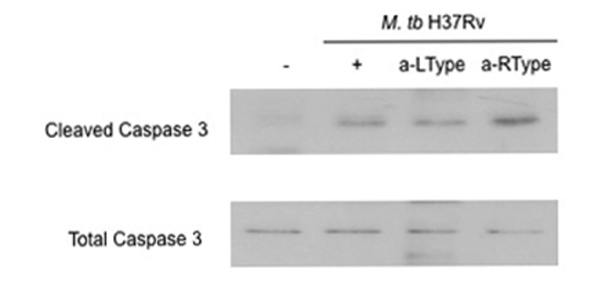

Supplement: Figure S7 — Blocking L-type and R-type VGCC in M. tb infected macrophages induces caspase 3 activation. Western blots of cleaved and total caspase 3 levels in mouse peritoneal macrophages infected with 1 MOI M. tb H37Rv for 24 h. In some groups L-type and R-type VGCC were blocked with antibody and the incubation continued for 24 h. Data are representative of two independent experiments. (0.06 MB TIF) [file pone.0005305.s007.tif]
